# A Disulfide Bond in the Membrane Protein IgaA Is Essential for Repression of the RcsCDB System

**DOI:** 10.3389/fmicb.2017.02605

**Published:** 2017-12-22

**Authors:** M. Graciela Pucciarelli, Leticia Rodríguez, Francisco García-del Portillo

**Affiliations:** ^1^Laboratorio de Patógenos Bacterianos Intracelulares, Departamento de Biotecnología Microbiana, Centro Nacional de Biotecnología-Consejo Superior de Investigaciones Científicas (CNB-CSIC), Madrid, Spain; ^2^Departamento de Biología Molecular, Universidad Autónoma de Madrid, Madrid, Spain; ^3^Centro de Biología Molecular Severo Ochoa-Consejo Superior de Investigaciones Científicas (CBMSO-CSIC), Madrid, Spain

**Keywords:** *Salmonella*, IgaA, periplasmic domain, cysteine, disulfide bond, RcsCDB

## Abstract

IgaA is an integral inner membrane protein that was discovered as repressor of the RcsCDB phosphorelay system in the intracellular pathogen *Salmonella enterica* serovar Typhimurium. The RcsCDB system, conserved in many members of the family *Enterobacteriaceae*, regulates expression of varied processes including motility, biofilm formation, virulence and response to envelope stress. IgaA is an essential protein to which, in response to envelope perturbation, the outer membrane lipoprotein RcsF has been proposed to bind in order to activate the RcsCDB phosphorelay. Envelope stress has also been reported to be sensed by a surface exposed domain of RcsF. These observations support a tight control of the RcsCDB system by RcsF and IgaA via mechanisms that, however, remain unknown. Interestingly, RcsF and IgaA have four conserved cysteine residues in loops exposed to the periplasmic space. Two non-consecutive disulfide bonds were shown to be required for RcsF function. Here, we report mutagenesis studies supporting the presence of one disulfide bond (C404-C425) in the major periplasmic loop of IgaA that is essential for repression of the RcsCDB phosphorelay. Our data therefore suggest that the redox state of the periplasm may be critical for the control of the RcsCDB system by its two upstream regulators, RcsF and IgaA.

## Introduction

The RcsCDB phosphorelay is a regulatory system conserved in most members of the family *Enterobacteriaceae* (Majdalani and Gottesman, 2007). A major role of this system is to monitor cell envelope stress, responding to alterations in outer membrane integrity and peptidoglycan structure (Farris et al., 2010; Evans et al., 2013; Konovalova et al., 2016). The tripartite RcsCDB system is atypical compared to most known phosphorelays, normally progressing from a sensor membrane protein to a cytosolic response regulator (Wolanin et al., 2002; Majdalani and Gottesman, 2005). In the RcsCDB system, the signal is transmitted from the sensor inner membrane protein RcsC to the intermediate membrane protein RcsD to end with phosphorylation of a conserved aspartate residue in the RcsB response regulator. The RcsCDB system controls expression of more than 40 genes involved in biofilm formation, synthesis of exopolysaccharide capsule, motility, and virulence among others (Hagiwara et al., 2003; Majdalani and Gottesman, 2007; Mariscotti and Garcia-del Portillo, 2009; Howery et al., 2016). The genes of the RcsCDB regulon were initially classified in those regulated exclusively by RcsB and, a second group including those involved in exopolysaccharide synthesis, which are controlled by RcsB and the co-regulator RcsA (Dierksen and Trempy, 1996; Navasa et al., 2013). Recent studies in *Escherichia coli* demonstrate that RcsB can heterodimerize with other co-regulatory proteins (Pannen et al., 2016). RcsB has also been shown to have a notable conformational dynamism (Casino et al., 2017), which could explain its capacity for providing different responses depending the phosphorylation status and the type and intensity of the stimulus (Mariscotti and Garcia-del Portillo, 2009; Latasa et al., 2012).

The RcsCDB system displays a feature conserved in most other regulatory networks, regarding its rapid response to the stress signal followed by a progressive decrease in activity once the bacterium adapts to the new environmental conditions (Gao and Stock, 2017). Of interest, these regulatory systems are “prepared to act” as denoted by the presence of all components of the signaling cascade even in the absence of stimulus. Thus, isogenic mutants of *Salmonella enterica* serovar Typhimurium (*S.* Typhimurium) displaying differences in the expression of RcsB target genes produce similar relative levels of the RcsC, RcsD, and RcsB proteins (Dominguez-Bernal et al., 2004).

Two important regulatory elements acting upstream of the RcsCDB system are the outer membrane lipoprotein RcsF and the integral inner membrane protein IgaA. RcsF was first reported as a lipoprotein that transmits a stress signal to the inner membrane sensor RcsC following cell envelope perturbations (Majdalani et al., 2005). IgaA was discovered as an integral inner membrane protein that contributes to attenuate the growth rate of *S.* Typhimurium inside eukaryotic cells (Cano et al., 2001). Subsequent studies revealed that the mucoid phenotype exhibited by a mutant bearing a R188H mutation in IgaA was linked to over-activation of the RcsCDB phosphorelay (Cano et al., 2002; Dominguez-Bernal et al., 2004). IgaA is predicted to have four transmembrane domains with the R188 residue located in one of the cytosolic domains (Dominguez-Bernal et al., 2004). Unlike the wild type IgaA protein, produced at constant levels in actively growing and resting bacteria, the R188H variant is unstable in stationary phase (Dominguez-Bernal et al., 2004). Although the loss of IgaA can be supported in non-growing bacteria, genetic evidence obtained in *S.* Typhimurium and *E. coli* demonstrates that *igaA* is an essential gene (Cano et al., 2002; Cho et al., 2014). Of note, IgaA become dispensable if the RcsCBD system is genetically inactivated (Cano et al., 2002). Moreover, loss-of-function mutations in the RcsCDB system are selected at high rate when attempting to replace the wild-type *igaA* gene by a null allele (Mariscotti and Garcia-Del Portillo, 2008). Altogether, these observations reveal a critical function of IgaA as a dedicated repressor of the RcsCDB phosphorelay in actively growing bacteria. Transcriptomic analyses also pointed to a major role of IgaA in fine-tuning the RcsCDB phosphorelay (Mariscotti and Garcia-del Portillo, 2009).

A recent study has provided the first insights into the mechanism by which the upstream regulators, RcsF and IgaA, could control activity of the RcsCDB phosphorelay (Cho et al., 2014). These authors showed that in steady-state growth conditions, RcsF is exposed in the external face of the outer membrane via interaction with OmpA and BamA, the major component of the β-barrel assembly machinery. Following peptidoglycan stress, RcsF fails to interact with OmpA/BamA and, as a result, retained in the periplasmic space. In this condition RcsF binds to the major periplasmic domain of IgaA to activate the RcsCDB phosphorelay (Cho et al., 2014). Based on the previous functional data obtained with IgaA, the RcsF-IgaA interaction must therefore alleviate the repression that IgaA exerts on the RcsCDB system in non-stimulatory conditions. Envelope stress can also be directed sensed by the surface-exposed domain of RcsF when defects in lipopolysaccharide structure occur (Konovalova et al., 2016).

RcsF has four conserved cysteines that form disulfide bonds (Leverrier et al., 2011). The formation of these disulfide bonds in RcsF depends on DsbC, the main disulfide isomerase, which together with the disulfide oxidase DsbA, control the formation and correct configuration of disulfide bonds (Denoncin and Collet, 2013). Disulfide bridges can play a structural role, as stable bonds, or; alternatively, contribute to catalysis by forming reversible disulfide bonds in the catalytic site (Denoncin et al., 2013). This latter case is exemplified by periplasmic oxidoreductases such as DsbA and DsbC. Whether the disulfide bonds of RcsF play a role in the interaction with IgaA is unknown.

In this study, we investigated the presence of disulfide bonds in IgaA, which contains four conserved cysteine residues in its major periplasmic domain. Our results are consistent with the presence of a disulfide bond in IgaA that is important for its function as repressor of the RcsCDB phosphorelay.

## Materials and Methods

### Bacterial Strains and Growth Conditions

The bacterial strains and plasmid used in this study are listed in Supplementary Table S1. Bacteria were cultured in Luria-Bertani (LB) broth at 37°C in shaking (150 rpm) conditions. To prepare material from mid-exponential cultures, the overnight culture was diluted 1:100 in fresh LB medium and collected at optical density (absorbance at 600 nm) of ∼0.2–0.3. The remaining culture was incubated for additional 18 h to obtain stationary phase cultures. When required, the medium was supplemented with ampicillin (50 μg/ml), kanamycin (30 μg/ml), tetracycline (10 μg/ml), or chloramphenicol (10 μg/ml).

### Mutagenesis of Periplasmic Cysteines

The mutagenesis was carried out with the Quick-change^TM^ site-directed mutagenesis kit from Stratagene, following manufacture recommendations. The oligonucleotides used for these procedures, including those degenerated introducing the desired point mutations, are listed in Supplementary Table S2. A copy of the *S.* Typhimurium wild-type *igaA* gene was cloned in the pBAD18 vector (plasmid pNG1062, Supplementary Table S1) and used as template for the mutagenesis kit to obtain pNG1062-derivate plasmids containing the mutant alleles (C404S, C425S, C498, and C504S), which were cloned in *E. coli* DH5α (Supplementary Table S1). The desired mutations were confirmed by sequencing. To generate the C404S–498S and C404S-C504S mutant alleles, a BlpI/BlpI fragment from the pLR1435 [pBAD18::*igaA*(C404S)] was used to replace the same region in pLR1438 [pBAD18::*igaA*(C498S)] and pLR1481 [pBAD18::*igaA*(C504S)] plasmids. The series of pNG1062-derivate plasmid (pBAD18 backbone) was transferred to the *S.* Typhimurium MD0835 strain [*igaA2*::KXX Δ(*apbE′-rcsC′*)], a mutant not producing IgaA and with an additional mutation in *rcsC* (Mariscotti and Garcia-Del Portillo, 2008). The production of the distinct IgaA variants with mutated cysteine residues was confirmed in the MD0835-derivate strains grown in LB-0.2% L-arabinose and subsequent analysis of total protein extracts by Western assay using anti-IgaA antibody (Cano et al., 2002).

### Generation of *S.* Typhimurium Strains with *igaA* Mutant Alleles (C404S, C425S, C498S, C504S) Disposed in the Chromosome

To transfer the *igaA* mutant alleles to the chromosome, they were first moved from the pBAD cloning vector to the pCVD442 suicide vector (Donnenberg and Kaper, 1991), which has the counter selectable marker *sacB*, using *E. coli* DH5α as host strain (Supplementary Table S1). Since we did not initially know whether the *igaA* mutant alleles could support viability (*igaA* is an essential gene in a *rcsCDB*^+^ background), the *igaA* mutant alleles were first moved to the chromosome of *S.* Typhimurium strain MD1446 (*igaA2*::KXX *zhf-6311*::Tn*10d*Tet *rcsC*::Mu*d*Q). This was done by conjugation using as donor *E. coli* SM10aaapir carrying the respective series of pCVD442 derivate plasmids. Loss of kanamycin resistance in the *S.* Typhimurium recipient strain was indicative of replacement of the *igaA* null allele (*igaA2*::KXX). The cysteine-defective *igaA* alleles were further transferred by P22 phage transduction to a clean wild-type background selecting by tetracycline resistance (flanking marker *zhf-6311*::Tn*10d*Tet). The *igaA* gene was PCR-amplified from all Tet^R^ transductants to confirm presence in the chromosome of the mutation in the codon of the corresponding cysteine residue. Whereas all single *igaA* mutants (C404S, C425S, C498S, and C504S) proved to support viability, no transductants were obtained attempting to transduce to a wild-type background the double mutant alleles C404S–C498S and C404S–C504S. This result was consistent with the viability test performed with the different *igaA* variants expressed from pBAD using different L-arabinose concentrations (see below, **Table 1**).

**Table 1:** Suppression of lethality associated to the *igaA*::Km null allele by ectopic expression of different IgaA variants.

	Number of Km^R^ transductants obtained with an *igaA*::Km P22 phage lysate							
% L-arabinose (w/v)	pBAD18 (empty vector)	*igaA*^+^ pBAD18	*igaA- C404S*	*igaA- C425S*	*igaA- C498S*	*igaA-C504S* pBAD18	*igaA-C404S- C498S* pBAD18	*igaA-C404S- C504S* pBAD18
0	0	0	0	0	0	0	0	0
0.002	0	17/24	0	0	0	0	0	0
0.02	0	1250/1440	389/369^∗^	437/384^∗^	728/726^∗^	384/390^∗^	0	0
0.2	0	5500/6000	505/454^∗^	393/360	716/720	408/412	484/500^∗^	318/286^∗^
2	0	10500/9000	687/612^∗^	716/717	970/1138	600/650	543/672^∗^	406/454^∗^
5	0	3200/3400	850	618/716	768/815	730	540/672^∗^	492/315^∗^

*^∗^These transductants were mucoid, denoting reduced repression of the RcsCDB system by the respective IgaA variant. No Km^R^ transductants bearing the igaA::Km null allele were obtained when using 2% glucose (w/v) instead of arabinose.*

### Western Blot Analyses

Preparation of protein extracts, electrophoresis and Western assay conditions using polyclonal rabbit anti-IgaA antibody were as described (Dominguez-Bernal et al., 2004).

### AMS Alkylation Assays

These assays were performed using the 4′-acetamido-4′-maleimidylstilbene-2,2′-disulfonic acid (AMS) reagent, as described (Jurado et al., 2006).

### β-galactosidase Assays

Levels of β-galactosidase derived from the *gmm*::*lacZ* transcriptional fusion were assayed as described by Miller, following the chloroform/SDS permeabilization procedure (Miller, 1972). For these assays, bacteria were grown in LB medium to mid-exponential phase (OD_600_ ∼ 0.2–0.3).

### Motility Assays

Motility of the different strains used was monitored by motility assays in soft agar plates, as described (Rosu et al., 2006).

### Statistical Analysis

Data were analyzed by one-way ANOVA using Prism version 5.0 (Graph-Pad Software). Differences in values with *P* < 0.05 were considered significant.

## Results

### IgaA Has Four Cysteines in the Periplasmic Domain Conserved in All Orthologs of Enteric Bacteria

**FIGURE 1:**
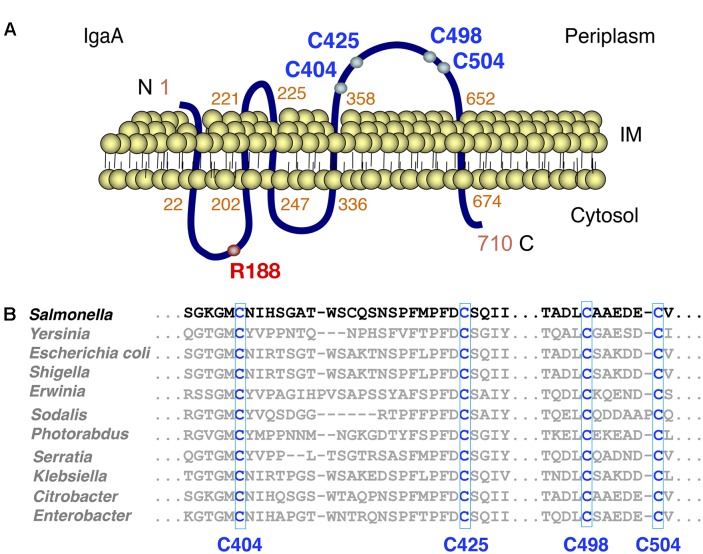
The integral inner membrane protein IgaA has four conserved periplasmic cysteines. **(A)** Predicted topology of the *Salmonella* Typhimurium IgaA protein of 710 amino acids length. The periplasmic cysteines C404, C425, C498, and C504 as well as the cytosolic R188 residue important for IgaA function, are shown. **(B)** Alignment of the periplasmic domain of distinct IgaA orthologs from the indicated members of the *Enterobacteriaceae* family. Alignment was performed with the Clustal Omega tool.

Our previous studies in *S.* Typhimurium showed that IgaA is an inner membrane protein produced at relatively constant levels in all growing conditions (Dominguez-Bernal et al., 2004). Programs that predict transmembrane helix regions and protein topology (THMM, SOSUI, TMPred, PredictProtein) indicate that the 710 amino acid protein IgaA has five transmembrane segments, resulting in two cytosolic domains (residues 22–202, 247–336), one small periplasmic loop (221–225) and one major periplasmic domain (residues 358–652) (**Figure 1A**). Mutations in specific residues of the two cytosolic loops (R188H, T191P, G262R) as well as in the periplasmic domain (L514P, L643P) impact negatively the capacity of IgaA to repress the RcsCDB phosphorelay (Dominguez-Bernal et al., 2004). We further noted that the periplasmic domain of *S.* Typhimurium IgaA has four cysteines, (C404, C425, C498, C504) highly conserved in IgaA orthologs found in distinct genera of enteric bacteria (**Figure 1B**). Based on this observation, we assessed whether these periplasmic cysteines could form disulfide bonds and play an important role in function.

**FIGURE 2:**
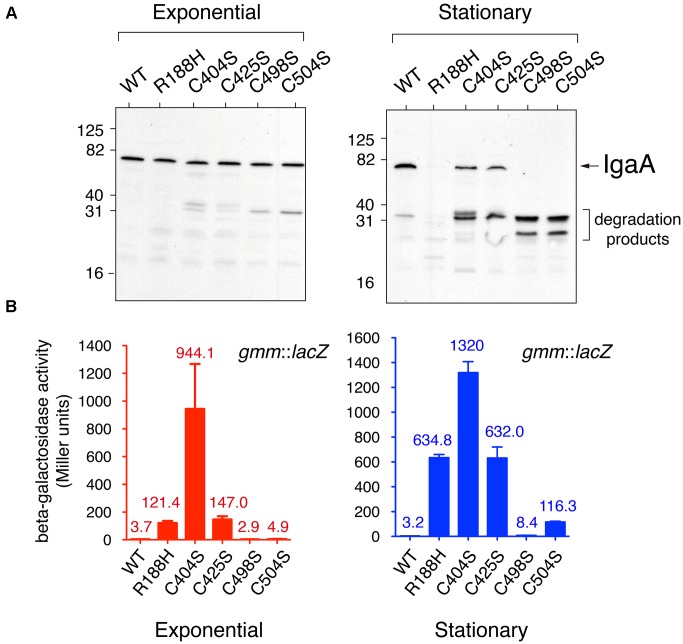
*Salmonella* Typhimurium IgaA variants in conserved cysteines show distinct capacities to repress the RcsCDB system. **(A)** IgaA was detected in western assays using samples obtained from bacteria grown in LB medium at 37°C to mid-exponential (OD_600_ = 0.2) or stationary (OD_600_ = 2.0) phase. Note the degradation of the IgaA cysteine variants when bacteria reach the stationary phase. The previously characterized IgaA variant R188H (Dominguez-Bernal et al., 2004) was used as control of protein unstable in stationary phase. Numbers on the left indicate the position of molecular weight markers (in kDa). **(B)** Activity of the RcsCDB system monitored with a g*mm*::*lacZ* reporter fusion in bacteria growing to exponential or stationary phases. *gmm* encodes an enzyme involved in synthesis of the colanic acid capsule and is positively regulated by the RcsCDB system. Data are the average and standard deviation of three independent experiments. The averages are shown as numbers on top of the respective bars.

To determine the role played by the C404, C425, C498, and C504 residues, we generated isogenic *S.* Typhimurium mutants lacking each of these cysteines. To this aim, we first introduced in the chromosome the corresponding *igaA* point mutant allele using as recipient an *igaA*::KXX *rcsC* strain, to subsequently pass the allele to a wild-type (*rcsCDB*^+^) genetic background by P22 phage transduction (see section “Material and Methods”). Importantly, none of the C404S, C425C, C498S, and C504S mutations affected protein stability in actively growing bacteria. Thus, in bacteria grown to exponential phase these IgaA variants were detected with similar levels than those of wild-type IgaA or the previously characterized R188H variant (**Figure 2A**) (Dominguez-Bernal et al., 2004). Nonetheless, all these four cysteine variants (C404S, C425S, C498S, and C504S) were unstable in stationary phase (**Figure 2A**). This phenomenon was reminiscent of that observed for other partially inactive variants such as R188H and L514P (Dominguez-Bernal et al., 2004). Therefore, we concluded that the elimination of any of the four conserved residues (C404, C425, C498, and C504) may lead to structural changes in IgaA that affect its stability when bacteria reach stationary phase. Considering the essentiality of IgaA linked to necessary repression of the RcsCDB system, all these cysteine variants were, however, expected to retain some partial function in growing bacteria.

### Elimination of the Periplasmic Cysteines (C404, C425, C498, and C504) of IgaA Results in Distinct De-repression Levels of the RcsCDB System

To determine the capacity of the IgaA cysteine variants to repress the RcsCDB phosphorelay, we monitored several phenotypic traits associated to the activity of this regulatory system. We included production of colanic acid capsule, which is positively controlled by RcsCDB; and, flagella production, negatively regulated by the system. In a first series of experiments, we measured in actively growing (exponential) and resting (stationary phase) bacteria the expression levels of *gmm* (*wcaH*), a gene encoding GDP-mannose mannosyl hydrolase, an enzyme involved in colanic acid capsule synthesis. The results showed that the C404S mutation caused partial de-repression of the RcsCDB system, even in actively growing bacteria when the protein remained stable (**Figures 2A,B**). Among the other mutants, a gradual variation in the level of RcsCDB activity was noted following the order C404S > C425S ∼ R188H > C504S > C498S > wild-type (**Figure 2B**). Interestingly, the levels of the RcsCDB system in stationary phase inferred from the *gmm*::*lacZ* reporter fusion increased only slightly despite the partial degradation observed for some of the IgaA mutant proteins such as C404S, C425S, C498S, or C504S (**Figures 2A,B**). These data are consistent with a major role of IgaA in repressing the RcsCDB that is critical only during active growth. These data also indicated that C404 and C425 are cysteines more important for function in comparison to C498 and C504.

**FIGURE 3:**
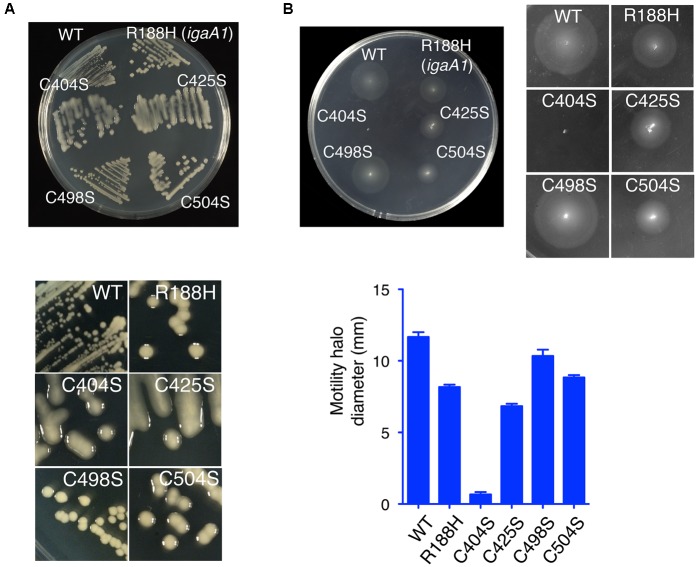
The periplasmic cysteines C404 and C425 are essential for the negative regulation that IgaA imposes over the RcsCDB phosphorelay. **(A)** Mucoid phenotype of strains producing the C404S, C425S, C504S, and R188H variants. **(B)** Motility test in soft agar. Note that the high repression of motility in the strain producing the C404S variant. A graphic showing motility halo diameter (average and standard deviation from three independent assays) is also depicted.

Additional phenotypic traits that were examined included the formation of mucoid colonies on plates (signal of capsule formation) and motility assays in soft agar plates. In agreement with the data obtained with the *gmm*(*wcaH*)::*lacZ* reporter fusion, the mutant producing IgaA-C404S was highly mucoid and non-motile (**Figures 3A,B**). For the rest of mutants, a gradual variation in RcsCDB activity was noted in the mucoidity and motility tests (**Figures 3A,B**), which in some cases were not completely matching the *gmm*::*lacZ* assays performed in liquid culture (**Figure 2B**). Thus, although the IgaA-C504S variant exhibited lower *gmm*::*lacZ* expression than R188H (**Figure 2B**), bacteria producing this C504S variant were slightly more mucoid on plates (**Figure 3A**). R188H, C425S, and C504S variants also displayed an intermediate phenotype in motility despite their variations in the *gmm*::*lacZ* assays or mucoidy on plates (**Figures 2B**, **3A,B**). Such discrepancies in the different tests may be influenced by the different growth conditions used -liquid culture vs. solid agar media plates-. Interestingly, mutations in defined RcsB residues alter the phosphorylation status of this regulator with consequences in either mucoidy or motility, but not in both phenotypic traits (Casino et al., 2017). Some of the mutations described here in the periplasmic cysteines of IgaA could result in distinct RcsB∼P/RcsB ratios, a hypothesis to be tested in future studies. Despite the minor phenotypic differences noted in IgaA for the R188H, C425S, and C504S variants; taken together, our data support that among the four periplasmic cysteines analyzed, C404 and C425 are residues with a more critical role for repression of the RcsCDB phosphorelay.

### IgaA Has One Disulfide Bond in the Periplasmic Domain

**FIGURE 4:**
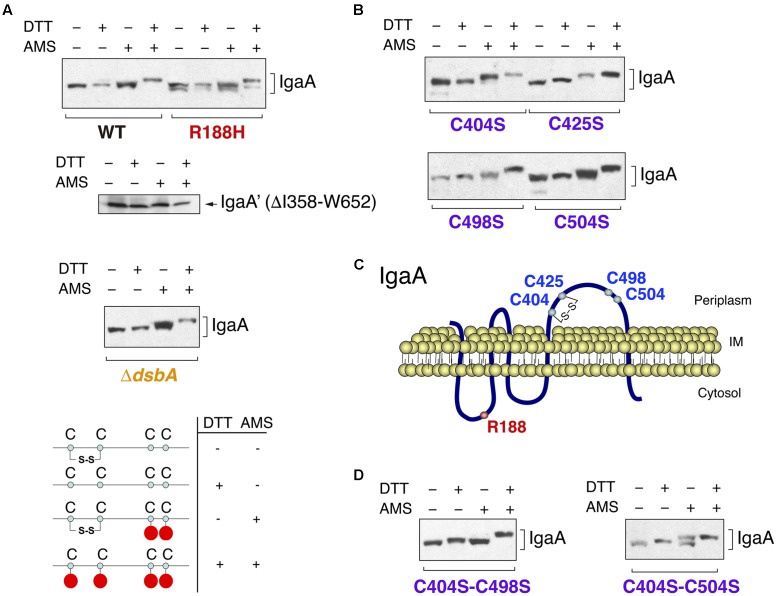
The periplasmic domain of IgaA has a C404–C425 disulfide bond essential for function that can be formed independently of the major disulfide oxidoreductase DsbA. **(A)** Alkylation assays with the 4′-acetamido-4′-maleimidylstilbene-2,2′-disulfonic acid (AMS) reagent reveal electrophoretic shifts in wild-type IgaA but not in a variant lacking the periplasmic domain (DI358-W652). Note the co-existence of distinct forms in the IgaA-R188H mutant and the lack of effect of a Δ*dsbA* mutation in the presence of IgaA forms with distinct electrophoretic mobility. A scheme is shown denoting the changes expected for DTT/AMS incubations for an example of one disulfide bond. **(B)** AMS alkylation assays in the IgaA variants C404S, C425S, C498S, and C504S. Note that the lack of C404 results in no major changes in electrophoretic mobility in the samples treated or not with DTT. **(C)** Proposed configuration of the C404–C425 disulfide bond in the periplasmic domain of IgaA. **(D)** Detection of non-native disulfide bridges in the C404S–C498S and C404S–C504S IgaA variants. Note the similarity of electrophoretic mobility of the non-alkylated and alkylated samples not treated with DTT (see text for details).

To elucidate whether the contribution of the periplasmic cysteines to IgaA function relies in the formation of disulfide bonds, we carried out alkylation experiments using the 4′-acetamido-4′-maleimidylstilbene-2,2′-disulfonic acid (AMS) reagent (Jurado et al., 2006; Denoncin et al., 2013). These experiments were performed in exponential phase (OD_600_ ∼0.2–0.3), in which all IgaA variants with mutated cysteines are stable (see **Figure 2A**). AMS is a maleimide compound that binds to free thiol groups. Thus, it is possible to differentiate the presence of a disulfide bond if changes in electrophoretic mobility are detected in samples incubated with AMS and previously treated or not with a reducing agent such as di-thio-threitol (DTT). When *S.* Typhimurium wild type cells were incubated in solutions with or without DTT and further treated or not with AMS, we detected four IgaA forms with distinct electrophoretic mobility (**Figure 4A**). This result proved the presence of at least one disulfide bond in IgaA.

Besides the four periplasmic cysteines, IgaA of *S.* Typhimurium has four additional cysteines that map in the first and second transmembrane regions (C14, C219, respectively), the second cytosolic domain (C259) and, the short cytosolic domain encompassing the C-terminal end of the protein (C697). To discard the contribution of these cysteines to the mobility shift displayed by wild-type IgaA in the alkylation assays, we generated a variant lacking the periplasmic domain (ΔI358-W652). This variant, unlike the full-length protein, did not exhibit electrophoretic shift in cells exposed to DTT and subsequently to AMS (**Figure 4A**). Therefore, none of the non-periplasmic cysteines of IgaA contribute to the formation of disulfide bonds. Interestingly, the different electrophoretic forms of IgaA observed in the AMS alkylation assays in wild-type bacteria were also detected in a Δ*dsbA* mutant (**Figure 4A**). This result indicated that the disulfide bond present in IgaA can be formed in the absence of the major disulfide oxidase DsbA.

To define the configuration of the periplasmic disulfide bond(s) inferred in wild-type IgaA, we next performed AMS alkylation assays in isogenic strains lacking each of the four conserved periplasmic cysteines. These assays revealed an important contribution of C404 in the formation of a disulfide bond. Thus, unlike the C425S, C498S, and C504S variants, the C404S variant did not migrate differently in the presence/absence of DTT (**Figure 4B**). Interestingly, the four variants in the conserved periplasmic cysteines (C404, C425, C498, and C504) showed differences in mobility when comparing the samples further exposed to AMS (**Figure 4B**). This result led us to consider the presence of one “native” C404–C425 disulfide bond and contrasting outcomes when one or the other cysteine residue is mutated. In the absence of C404, we only see possible a non-native C498–C504 bond with minor consequences in electrophoretic migration as evidenced by the similar behavior of -/+ DTT samples (**Figure 4B**). In contrast, the lack of C425 might favor the formation of non-native C404–C498 or C404–C504 disulfide bonds, which could explain the clear electrophoretic shift observed in the -/+ DTT samples of the C425 mutant (**Figure 4B**). The more important role assigned to C404 and C425 in comparison to C498 and C504 agrees with the much higher RcsCDB activity registered when any of these two cysteines (C404 or C425) is lacking (**Figures 2B**, **3A,B**).

Since the IgaA-R188H variant is only partially efficient in repressing the RcsCDB system (Cano et al., 2002; Dominguez-Bernal et al., 2004), we sought to determine whether this mutation could have effect on the C404–C425 disulfide bond. Unexpectedly, we detected molecules with distinct redox state that co-exist in this IgaA-R188H variant (**Figure 4A**). Thus, two forms with distinct electrophoretic mobility were clearly distinguishable in samples non-treated with DTT or the alkylating agent AMS (**Figure 4A**). Interestingly, DTT moved these two forms to a single one that was coincident to the fully reduced form of wild type IgaA (see **Figure 4A**). This result demonstrated that a mutation such as R188H mapping in a cytosolic loop can affect proper formation of a disulfide bond in the major periplasmic loop.

Taken together, these data are consistent with a model in which the function of IgaA as repressor of the RcsCDB system depends on the redox state of its periplasmic domain, which in its functional conformation may involve the formation of a C404–C425 disulfide bond (**Figure 4C**).

### The Lack of the C404–C425 Disulfide Bond or the Formation of Alternative Non-native Disulfide Bonds Are Lethal in a RcsCDB^+^ Background

To further support the essential role that the C404–C425 disulfide bond plays in repression of the RcsCDB phosphorelay, we generated IgaA double mutants. Our aim was to affect the native bridge (C404–C425) and to simultaneously impair alternative non-native disulfide bonds predicted by the AMS alkylation assays and involving either C498 or C504 (**Figure 4B**). Therefore, the new IgaA variants were C404S–C498S and C404S-C504S. The AMS alkylation assays revealed no difference in migration between the non-alkylated and alkylated samples not exposed to DTT for any of these two double mutants (**Figure 4D**). This result, much more evident in the case of the C404S–C498S variant, supported the absence of free periplasmic cysteines and, therefore, the presence a “non-native” disulfide bond (C425–C504). In the C404S-C504S variant, the alkylation assays revealed two bands in the case of the samples non-treated with DTT and exposed to AMS (**Figure 4D**), implying the co-existence of molecules with and without a non-native C425–C498 disulfide bond. This phenomenon resembled at some extent the co-existence of molecules with different redox state observed for the IgaA-R188H variant (**Figure 4A**).

We next assessed whether the non-native C425–C498 or C425–C504 bonds could provide functionality to the protein. To this aim, we analyzed the capacity of the different IgaA variants to suppress lethality associated to the presence of an *igaA*::Km null allele in an RcsCDB^+^ genetic background (Cano et al., 2002; Mariscotti and Garcia-Del Portillo, 2008). The number of *igaA*::Km transductants obtained in strains bearing inducible pBAD18 vectors expressing the different IgaA variants was determined in the absence/presence of the inducer, L-arabinose. As a negative control, we used a strain with the pBAD18 empty vector, for which no Km^R^ transductants carrying the *igaA*:.km null allele were obtained regardless the absence/presence of inducer.

These assays showed that the C404S–C498S and C404S–C504S variants were not capable of suppressing the lethality associated to the *igaA*::km mutant when induced at 0.02% L-arabinose, a concentration sufficient to prevent lethality by any of the single mutants lacking each of the conserved cysteine residues (**Table 1**). Furthermore, we observed that when suppressing lethality at higher arabinose concentrations, all transductans were mucoid (**Table 1**). This result was indicative of the extremely limited function of these double C404S–C498S and C404S–C504S variants, even when produced at high levels. Therefore, the non-native C425-C498 and C425–C504 disulfide bonds are not optimal to provide function to IgaA.

## Discussion

In this study, we have examined the role in function of four conserved cysteines located in the periplasmic domain of the RcsCDB repressor IgaA. Our data prove the presence in the native protein of a periplasmic disulfide bond in the configuration C404-C425. The alkylation experiments suggest that a C404S mutation renders the protein unable to form any stable disulfide bond, a scenario slightly difference to that of the C425S mutation, in which non-native C404–C498 or C404–C504 bonds were inferred. This interpretation agrees with the high de-repression of the RcsCDB phosphorelay observed in *S.* Typhimurium strains expressing the IgaA-C404S variant. Importantly, our data discarded any compensatory role in function for the non-native disulfide bonds C404–C498 or C404-C504 that apparently occur in the C425S mutant. Therefore, not all disulfide bonds capable of forming in the periplasmic domain support equally IgaA function as RcsCDB repressor. A similar conclusion was reached for RcsF of *E. coli*, which has two not functionally equivalent disulfide bonds (Leverrier et al., 2011; Rogov et al., 2011), with one of them proposed to be more relevant for function (Leverrier et al., 2011).

The alkylation experiments performed with the Δ*dsbA* mutant discarded an absolute requirement of this disulfide oxidase for formation of the C404–C425 bond. Thus, different IgaA forms with distinct electrophoretic mobility were detected in this Δ*dsbA* mutant depending the presence/absence of DTT and/or AMS. This result opens the possibility of IgaA being recognized by alternative disulfide oxidases. Besides the pair DsbA/DsbB, *S.* Typhimurium encodes the paralogs DsbL and DsbI (Lin et al., 2009) and has an additional DsbA paralog, SrgA, encoded in the virulence plasmid (Bouwman et al., 2003). Interestingly, SsaC, a virulence-related protein related to the type III secretion system encoded in the *Salmonella* pathogenicity island 2 (SPI-2), was shown to be oxidized indistinctly by DsbA or SrgA (Miki et al., 2004). Further experiments are needed to confirm whether IgaA could be recognized by any of these alternative disulfide oxidases.

An unexpected finding of our study was the co-existence of IgaA molecules with different redox states. This situation, not observed for the wild-type IgaA, was evident for the R188H and the C404S–C504S variants (**Figures 4A,D**). Such behavior may reflect conformational plasticity in the periplasmic domain of IgaA, which may be altered at some degree when mutations in key residues important for function are introduced. Importantly, the observations with the R188H mutation proved that disrupting a key residue for function in the cytosolic side can have consequences in the structure of the periplasmic loop. This evidence points to signal transmission within the IgaA molecule from one to other side of the inner membrane.

Another aspect of interest found in the study was the instability of all IgaA variants lacking the conserved periplasmic cysteines when bacteria reached stationary phase. Proteolysis in stationary phase is not a common process. Some of the few cases known include YfgM and the formate dehydrogenase subunit FdoH, two substrates of the cytosolic FtsH protease (Westphal et al., 2012; Bittner et al., 2015). Based on our findings with the mutated IgaA variants, it is tempting to speculate on a protease that could monitor IgaA for proper folding in stationary phase. Noteworthy, mass spectrometry analyses revealed RcsF and the protease DegP as partners interacting with the periplasmic domain of IgaA, (Cho et al., 2014). Whether DegP monitors correct folding of the IgaA periplasmic domain requires further experiments.

An important feature of all cysteine IgaA variants is that they were fully stable in actively growing bacteria (**Figure 2**), a condition in which de-repression of the RcsCDB system (measured by the *gmm*::*lacZ* fusion) was evident in some cases as those of C404S and C425S mutations (**Figure 2B**). This experimental evidence supports a mode of action of IgaA involving a C404–C425 disulfide bond, absolutely essential for repressing the RcsCDB system.

The gradation observed in the phenotypic assays involving mucoidity and motility and the discrepancy found for some of the IgaA variants tested (R188H, C425S, C504S), may reflect a functional link between IgaA and the conformational plasticity recently reported for RcsB (Casino et al., 2017). RcsB plasticity, which is directly linked to its phosphorylation status, could allow the bacteria to perceive many signals with intensities of the RcsCDB phosphorelay previously adjusted by IgaA. This idea of a correspondence between RcsB phosphorylation and IgaA function is now testable, for example by measuring phosphorylation status of RcsB in the different cysteine variants of IgaA reported here. We should also not discard other factors contributing to the role that IgaA has in shaping the RcsB regulon.

Another point of interest raised by our data accounts for the interaction recently reported for RcsF and IgaA as an event triggering the activation of the RcsCDB phosphorelay (Cho et al., 2014). Disulfide bonds are favored in oxidative environments as the periplasm (Denoncin and Collet, 2013; Goemans et al., 2014), and it is in this environment where the hypothetical RcsF-IgaA interaction takes place. Whether this interaction is influenced by the conserved cysteines of each partner is unknown and future studies directed to test the effect of cysteine mutations or the presence/absence of the C404–C425 disulfide bond could be therefore of much interest.

## Conclusion

Our work provides evidence for the essential role played by one disulfide bond (C404–C425) of IgaA in its function as attenuator of the RcsCDB phosphorelay. Given the conservation of these two periplasmic cysteines in all IgaA orthologs known in enteric bacteria, the data reported here support an important structural role for this disulfide bond, probably facilitating an active conformation to the major periplasmic domain.

## Author Contributions

Experimental design, methodology and investigation: MP and LR. Conceptualization and supervision: FG-dP. Writing – original draft: MP, LR, and FG-dP. Writing – reviewing and editing: FG-dP.

## Conflict of Interest Statement

The authors declare that the research was conducted in the absence of any commercial or financial relationships that could be construed as a potential conflict of interest.

## References

[B1] Bittner L. M. Westphal K. Narberhaus F. (2015). Conditional proteolysis of the membrane protein Yfgm by the Ftsh protease depends on a novel n-terminal degron. *J. Biol. Chem.* 290 19367–19378. 10.1074/jbc.M115.648550 26092727 PMC4521054

[B2] Bouwman C. W. Kohli M. Killoran A. Touchie G. A. Kadner R. J. Martin N. L. (2003). Characterization of SrgA, a *Salmonella enterica* serovar typhimurium virulence plasmid-encoded paralogue of the disulfide oxidoreductase dsba, essential for biogenesis of plasmid-encoded fimbriae. *J. Bacteriol.* 185 991–1000. 10.1128/JB.185.3.991-1000.2003 12533475 PMC142830

[B3] Cano D. A. Dominguez-Bernal G. Tierrez A. Garcia-Del Portillo F. Casadesus J. (2002). Regulation of capsule synthesis and cell motility in *Salmonella enterica* by the essential gene igaA. *Genetics* 162 1513–1523. 12524328 10.1093/genetics/162.4.1513 PMC1462382

[B4] Cano D. A. Martinez-Moya M. Pucciarelli M. G. Groisman E. A. Casadesus J. Garcia-Del Portillo F. (2001). *Salmonella enterica* serovar Typhimurium response involved in attenuation of pathogen intracellular proliferation. *Infect. Immun.* 69 6463–6474. 10.1128/IAI.69.10.6463-6474.2001 11553591 PMC98782

[B5] Casino P. Miguel-Romero L. Huesa J. Garcia P. Garcia-Del Portillo F. Marina A. (2017). Conformational dynamism for DNA interaction in the *Salmonella* RcsB response regulator. *Nucleic Acids Res.* 10.1093/nar/gkx1164 [Epub ahead of print]. 29186528 PMC5758874

[B6] Cho S. H. Szewczyk J. Pesavento C. Zietek M. Banzhaf M. Roszczenko P. (2014). Detecting envelope stress by monitoring beta-barrel assembly. *Cell* 159 1652–1664. 10.1016/j.cell.2014.11.045 25525882

[B7] Denoncin K. Collet J. F. (2013). Disulfide bond formation in the bacterial periplasm: major achievements and challenges ahead. *Antioxid. Redox Signal.* 19 63–71. 10.1089/ars.2012.4864 22901060 PMC3676657

[B8] Denoncin K. Nicolaes V. Cho S. H. Leverrier P. Collet J. F. (2013). Protein disulfide bond formation in the periplasm: determination of the in vivo redox state of cysteine residues. *Methods Mol. Biol.* 966 325–336. 10.1007/978-1-62703-245-2_20 23299744

[B9] Dierksen K. P. Trempy J. E. (1996). Identification of a second RcsA protein, a positive regulator of colanic acid capsular polysaccharide genes, in *Escherichia coli*. *J. Bacteriol.* 178 5053–5056. 10.1128/jb.178.16.5053-5056.1996 8759878 PMC178297

[B10] Dominguez-Bernal G. Pucciarelli M. G. Ramos-Morales F. Garcia-Quintanilla M. Cano D. A. Casadesus J. (2004). Repression of the RcsC-YojN-RcsB phosphorelay by the IgaA protein is a requisite for *Salmonella* virulence. *Mol. Microbiol.* 53 1437–1449. 10.1111/j.1365-2958.2004.04213.x 15387821

[B11] Donnenberg M. S. Kaper J. B. (1991). Construction of an eae deletion mutant of enteropathogenic *Escherichia coli* by using a positive-selection suicide vector. *Infect. Immun.* 59 4310–4317. 1937792 10.1128/iai.59.12.4310-4317.1991 PMC259042

[B12] Evans K. L. Kannan S. Li G. De Pedro M. A. Young K. D. (2013). Eliminating a set of four penicillin binding proteins triggers the Rcs phosphorelay and Cpx stress responses in *Escherichia coli*. *J. Bacteriol.* 195 4415–4424. 10.1128/JB.00596-13 23893115 PMC3807471

[B13] Farris C. Sanowar S. Bader M. W. Pfuetzner R. Miller S. I. (2010). Antimicrobial peptides activate the Rcs regulon through the outer membrane lipoprotein RcsF. *J. Bacteriol.* 192 4894–4903. 10.1128/JB.00505-10 20675476 PMC2944553

[B14] Gao R. Stock A. M. (2017). Quantitative kinetic analyses of shutting off a two-component system. *mBio* 8:e00412-17. 10.1128/mBio.00412-17 28512092 PMC5433096

[B15] Goemans C. Denoncin K. Collet J. F. (2014). Folding mechanisms of periplasmic proteins. *Biochim. Biophys. Acta* 1843 1517–1528. 10.1016/j.bbamcr.2013.10.014 24239929

[B16] Hagiwara D. Sugiura M. Oshima T. Mori H. Aiba H. Yamashino T. (2003). Genome-wide analyses revealing a signaling network of the RcsC-YojN-RcsB phosphorelay system in *Escherichia coli*. *J. Bacteriol.* 185 5735–5746. 10.1128/JB.185.19.5735-5746.2003 13129944 PMC193970

[B17] Howery K. E. Clemmer K. M. Rather P. N. (2016). The Rcs regulon in *Proteus mirabilis*: implications for motility, biofilm formation, and virulence. *Curr. Genet.* 62 775–789. 10.1007/s00294-016-0579-1 26936153

[B18] Jurado P. De Lorenzo V. Fernandez L. A. (2006). Thioredoxin fusions increase folding of single chain Fv antibodies in the cytoplasm of *Escherichia coli*: evidence that chaperone activity is the prime effect of thioredoxin. *J. Mol. Biol.* 357 49–61. 10.1016/j.jmb.2005.12.058 16427080

[B19] Konovalova A. Mitchell A. M. Silhavy T. J. (2016). A lipoprotein/beta-barrel complex monitors lipopolysaccharide integrity transducing information across the outer membrane. *Elife* 5:e15276. 10.7554/eLife.15276 27282389 PMC4942254

[B20] Latasa C. Garcia B. Echeverz M. Toledo-Arana A. Valle J. Campoy S. (2012). *Salmonella* biofilm development depends on the phosphorylation status of RcsB. *J. Bacteriol.* 194 3708–3722. 10.1128/JB.00361-12 22582278 PMC3393492

[B21] Leverrier P. Declercq J. P. Denoncin K. Vertommen D. Hiniker A. Cho S. H. (2011). Crystal structure of the outer membrane protein RcsF, a new substrate for the periplasmic protein-disulfide isomerase DsbC. *J. Biol. Chem.* 286 16734–16742. 10.1074/jbc.M111.224865 21454485 PMC3089515

[B22] Lin D. Kim B. Slauch J. M. (2009). DsbL and DsbI contribute to periplasmic disulfide bond formation in *Salmonella enterica* serovar Typhimurium. *Microbiology* 155 4014–4024. 10.1099/mic.0.032904-0 19797361 PMC2889420

[B23] Majdalani N. Gottesman S. (2005). The Rcs phosphorelay: a complex signal transduction system. *Annu. Rev. Microbiol.* 59 379–405. 10.1146/annurev.micro.59.050405.101230 16153174

[B24] Majdalani N. Gottesman S. (2007). Genetic dissection of signaling through the Rcs phosphorelay. *Methods Enzymol.* 423 349–362. 10.1016/S0076-6879(07)23016-2 17609140

[B25] Majdalani N. Heck M. Stout V. Gottesman S. (2005). Role of RcsF in signaling to the Rcs phosphorelay pathway in *Escherichia coli*. *J. Bacteriol.* 187 6770–6778. 10.1128/JB.187.19.6770-6778.2005 16166540 PMC1251585

[B26] Mariscotti J. F. Garcia-Del Portillo F. (2008). Instability of the *Salmonella* RcsCDB signalling system in the absence of the attenuator IgaA. *Microbiology* 154 1372–1383. 10.1099/mic.0.2007/015891-0 18451046

[B27] Mariscotti J. F. Garcia-del Portillo F. (2009). Genome expression analyses revealing the modulation of the *Salmonella* Rcs regulon by the attenuator IgaA. *J. Bacteriol.* 191 1855–1867. 10.1128/JB.01604-08 19124574 PMC2648367

[B28] Miki T. Okada N. Danbara H. (2004). Two periplasmic disulfide oxidoreductases, DsbA and SrgA, target outer membrane protein SpiA, a component of the *Salmonella* pathogenicity island 2 type III secretion system. *J. Biol. Chem.* 279 34631–34642. 10.1074/jbc.M402760200 15169785

[B29] Miller J. H. (1972). *Experiments in Molecular Genetics.* Cold Spring Harbor, NY: Cold Spring Harbor Laboratory.

[B30] Navasa N. Rodriguez-Aparicio L. Ferrero M. A. Monteagudo-Mera A. Martinez-Blanco H. (2013). Polysialic and colanic acids metabolism in *Escherichia coli* K92 is regulated by RcsA and RcsB. *Biosci. Rep.* 33:e00038. 10.1042/BSR20130018 23607330 PMC3673037

[B31] Pannen D. Fabisch M. Gausling L. Schnetz K. (2016). Interaction of the RcsB response regulator with auxiliary transcription regulators in *Escherichia coli*. *J. Biol. Chem.* 291 2357–2370. 10.1074/jbc.M115.696815 26635367 PMC4732218

[B32] Rogov V. V. Rogova N. Y. Bernhard F. Lohr F. Dotsch V. (2011). A disulfide bridge network within the soluble periplasmic domain determines structure and function of the outer membrane protein RcsF. *J. Biol. Chem.* 286 18775–18783. 10.1074/jbc.M111.230185 21471196 PMC3099694

[B33] Rosu V. Chevance F. F. Karlinsey J. E. Hirano T. Hughes K. T. (2006). Translation inhibition of the *Salmonella fliC* gene by the *fliC* 5′ untranslated region, *fliC* coding sequences, and FlgM. *J. Bacteriol.* 188 4497–4507. 10.1128/JB.01552-05 16740956 PMC1482935

[B34] Westphal K. Langklotz S. Thomanek N. Narberhaus F. (2012). A trapping approach reveals novel substrates and physiological functions of the essential protease FtsH in *Escherichia coli*. *J. Biol. Chem.* 287 42962–42971. 10.1074/jbc.M112.388470 23091052 PMC3522291

[B35] Wolanin P. M. Thomason P. A. Stock J. B. (2002). Histidine protein kinases: key signal transducers outside the animal kingdom. *Genome Biol.* 3:REVIEWS3013. 12372152 10.1186/gb-2002-3-10-reviews3013 PMC244915

