# Supplementary material for: A Disulfide Bond in the Membrane Protein IgaA Is Essential for Repression of the RcsCDB System
Source: Front Microbiol. 2017 Dec 22;8:2605. doi: 10.3389/fmicb.2017.02605 (PMC5744062; doi:10.3389/fmicb.2017.02605)
Supplement: Supplementary file 1 [file Table_1.PDF]

SUPPLEMENTARY MATERIAL

A disulfide bond in the membrane protein IgaA is essential for repression of the RcsCDB system

M. Graciela Pucciarelli, Leticia Rodríguez and Francisco García-del Portillo

Table S1. *S.* Typhimurium/*E. coli* strains and plasmids used in this study

Table S2. Primer oligonucleotides used in this study.

Table S1. *S.* Typhimurium/*E. coli* strains and plasmids used in this study

Bacterial strain/ plasmid	Relevant genotype	Source/ reference
S. Typhimurium		
SL1344	*hisG64,* virulent strain	(Hoiseth and Stocker, 1981)
SV4450	SL1344 *igaA1* (R188H)	(Cano et al., 2002)
MD0835	SL1344 *igaA2*::KXX Δ(*apbE'-rcsC'*)	(Mariscotti and Garcia-Del Portillo, 2008)
SV4390	SL1344 *igaA2*::KXX *zhf-6311*::Tn*10d*Tet *rcsC*::Mu*d*Q	This study
MD1463	SL1344 *igaA* (C404S) *zhf-6311*::Tn*10d*Tet	This study
MD1459	SL1344 *igaA* (C404S) *zhf-6311*::Tn*10d*Tet *rcsC*::Mu*d*Q	This study
MD1494	SL1344 *igaA* (C425S) *zhf-6311*::Tn*10d*Tet	This study
MD1491	SL1344 *igaA* (C425S) *zhf-6311*::Tn*10d*Tet *rcsC*::Mu*d*Q	This study
MD1467	SL1344 *igaA* (C498S) *zhf-6311*::Tn*10d*Tet	This study
MD1460	SL1344 *igaA* (C498S) *zhf-6311*::Tn*10d*Tet *rcsC*::Mu*d*Q	This study
MD1493	SL1344 *igaA* (C504S) *zhf-6311*::Tn*10d*Tet	This study
MD1492	SL1344 *igaA* (C504S) *zhf-6311*::Tn*10d*Tet *rcsC*::Mu*d*Q	This study
MD1769	SL1344 *igaA* (C404S-C498S) *zhf-6311*::Tn*10d*Tet *rcsC*::Mu*d*Q	This study
MD1770	SL1344 *igaA* (C404S-C504S) *zhf-6311*::Tn*10d*Tet *rcsC*::Mu*d*Q	This study
MD1757	SL1344 *gmm*::*lacZ igaA* (C402S)	This study
MD1758	SL1344 *gmm*::*lacZ igaA* (C425S)	This study
MD1759	SL1344 *gmm*::*lacZ igaA* (C498S)	This study
MD1760	SL1344 *gmm*::*lacZ igaA* (C504S)	This study
MD4370	SL1344 Δ*dsbA*	This study

E. coli		
CC118λpir	*Δ(ara-leu) araD ΔlacX74 galE galK phoA20 thi-1 rpsE rpoB argE* (Am) *recA1 λpir*	(Herrero et al., 1990)
SM10λpir	*thi thr leu tonA lacY supE recA*::RP4-2-Tc::Mu Km λpir	(Simon, 1983)
JM110	*rpsL thr leu thi lacY galK galT ara tonA tsx dam dcm glnV44* Δ(*lac-proAB*) e14- [F' *traD*36 *proAB*$^+$ *lacI*^{q} *lacZ*ΔM15] *hsdR*17($r_K^- m_K^+$)	Stratagene
MD1433	JM110 / pNG1062 (pBAD18::*igaA*$^+$)	This study
DH5α	F$^-$ *endA1 glnV44 thi-1 recA1 relA1 gyrA96 deoR nupG purB20* φ80d*lacZ*ΔM15 Δ(*lacZYA-argF*)U169, hsdR17($r_K^- m_K^+$), λ$^-$	(Meselson and Yuan, 1968)
MD1435	DH5α / pLR1435 [pBAD18::*igaA*(C404S)]	This study
MD1451	DH5α / pLR1451 [pCVD442::*igaA*(C404S)]	This study
MD1479	DH5α / pLR1479 [pBAD18::*igaA*(C425S)]	This study
MD1483	DH5α / pLR1483 [pCVD442::*igaA*(C425S)]	This study
MD1438	DH5α / pLR1438 [pBAD18::*igaA*(C498S)]	This study
MD1449	DH5α / pLR1449 [pCVD442::*igaA*(C498S)]	This study
MD1481	DH5α / pLR1481 [pBAD18::*igaA*(C504S)]	This study
MD1484	DH5α / pLR1484 [pCVD442::*igaA*(C504S)]	This study
MD1763	DH5α / pLR1763 [pBAD18::*igaA* (C404S-C498S)]	This study
MD1765	DH5α / pLR1765 [pCVD442::*igaA*(C404S-C498S)]	This study
MD1764	DH5α / pLR1764 [pBAD18::*igaA* (C404S-C504S)]	This study
MD1766	DH5α / pLR1766 [pCVD442::*igaA*(C404S-C504S)]	This study
Plasmids		
pBAD18	Amp^R, expression vector (L-arabinose inducible)	(Guzman et al., 1995)
pCVD442	Amp^R, suicide vector containing the counter selectable marker *sacB*	(Donnenberg and Kaper, 1991)

Table S2. Primer oligonucleotides used in this study

Name	Sequence (5’ – 3’)	Purpose
IgaAC425S-1	GAA CGG CAT AAA CGG TGA GTT AGA C	C425 mutagenesis
IgaAC425S-2	GAT TCC TCG CAA ATC ATC TGG AAT GAC	C425 mutagenesis
C425UP	GCC GTT CGA TTC CTC GCA AAT CAT CTG G	C425 mutagenesis
C425RP	CCA GAT GAT TTG CGA GGA ATC GAA CGG C	C425 mutagenesis
IgaAC504S-1	ACT CGT CTT CCG CCG CGC ACA AAT	C504 mutagenesis
IgaAC504S-2	CCG TGC GTC TGA AAA ATG CGC TGG T	C504 mutagenesis
C504UP	GGC GGA AGA CGA GTC CGT GCG TCT G	
C504RP	CAG ACG CAC GGA CTC GTC TTC CGC C	
IgaAC498S-1	GAC AAA TCA GCG GTT TTC AGA ACA ATA TCG CC	C498 mutagenesis
IgaAC498S-2	CGC GGC GGA AGA CGA GTG CGT G	C498 mutagenesis
IgaAC404S-1	GAC ATT CCT TTA CCGCTC AGG TGC AGC GT	C404 mutagenesis
IgaAC404S-2	TAA TAT TCA TTC CGG CGC GAC CTG G	C404 mutagenesis
KO dsbA Fw	TCG GAG AGA GTT GAT CAT GAA AAA GAT TTG GCT GGC GCT GGT GTA GGC TGG AGC TGC TTC	mutant Δ*dsbA*
KO dsbA Rv	CCG GCG TTC TTT TTA TTT TTT ATC AAC CAA ATA TTT CAC AAT TCC GGG GAT CCG TCG ACC	mutant Δ*dsbA*
FL dsbA Fw	TTA CAA TTA ACG CCA ATG TAT TAA TCG GAG AGA GAG TTG ATC	verification Δ*dsbA*
FL dsbA Rv	AAC ATC TTA TAA AAA CGC CGG TCA GTG ACC GGC GTT CTT T	verification Δ*dsbA*

References

Cano, D.A., Dominguez-Bernal, G., Tierrez, A., Garcia-Del Portillo, F., and Casadesus, J. (2002). Regulation of capsule synthesis and cell motility in Salmonella enterica by the essential gene igaA. *Genetics* 162, 1513-1523.

Donnenberg, M.S., and Kaper, J.B. (1991). Construction of an eae deletion mutant of enteropathogenic Escherichia coli by using a positive-selection suicide vector. *Infect Immun* 59, 4310-4317.

Guzman, L.M., Belin, D., Carson, M.J., and Beckwith, J. (1995). Tight regulation, modulation, and high-level expression by vectors containing the arabinose PBAD promoter. *J Bacteriol* 177, 4121-4130.

Herrero, M., De Lorenzo, V., and Timmis, K.N. (1990). Transposon vectors containing non-antibiotic resistance selection markers for cloning and stable chromosomal insertion of foreign genes in gram-negative bacteria. *J Bacteriol* 172, 6557-6567.

Hoiseth, S.K., and Stocker, B.A. (1981). Aromatic-dependent Salmonella typhimurium are non-virulent and effective as live vaccines. *Nature* 291, 238-239.

Mariscotti, J.F., and Garcia-Del Portillo, F. (2008). Instability of the Salmonella RcsCDB signalling system in the absence of the attenuator IgaA. *Microbiology* 154, 1372-1383.

Meselson, M., and Yuan, R. (1968). DNA restriction enzyme from E. coli. *Nature* 217, 1110-1114.

Simon, R.P., U.; Pühler, A. (1983). A Broad Host Range Mobilization System for In Vivo Genetic Engineering: Transposon Mutagenesis in Gram Negative Bacteria. *Nature Biotechnology* 1, 784-791.
